# Recovery of Zinc and Iron from Steel Mill Dust—An Overview of Available Technologies

**DOI:** 10.3390/ma15124127

**Published:** 2022-06-10

**Authors:** Yang Xue, Xiansheng Hao, Xiaoming Liu, Na Zhang

**Affiliations:** 1 State Key Laboratory of Advanced Metallurgy, University of Science and Technology Beijing, Beijing 100083, China; cdxueyang@163.com; 2 School of Metallurgical and Ecological Engineering, University of Science and Technology Beijing, Beijing 100083, China; h18963760674@163.com; 3 Beijing Key Laboratory of Materials Utilization of Nonmetallic Minerals and Solid Wastes, National Laboratory of Mineral Materials, School of Materials Science and Technology, China University of Geosciences, Beijing 100083, China

**Keywords:** steel mill dust, zinc, recovery, hydrometallurgy, pyrometallurgy

## Abstract

The global shortage of zinc mines makes the extraction of zinc from zinc-containing wastes a hot research topic. Most kinds of steel mill dust (SMD) cannot be directly returned to the ironmaking and steelmaking processes due to their zinc content. A large amount of SMD produced during steelmaking has become a major challenge for steel plants due to environmental pollution, health issues and land scarcity. Lots of processes for recovering metals from SMD have been developed to comprehensively utilize them and solve these environmental problems. Zinc in SMD can be recovered by these technologies, and the residue can be used as raw material for ironmaking. In this work, the sources and characteristics of SMD from different processes were analyzed firstly. Then, the mechanisms of physical, hydrometallurgical, pyrometallurgical and pyrometallurgy–hydrometallurgy combined processes for SMD disposal are presented, and these methods are compared in terms of energy consumption, process complexity and industrial application. Finally, suggestions and prospects for utilization of SMD are put forward.

## 1. Introduction

Zinc metal is widely used in various fields of social life, and its social consumption is only inferior to copper and aluminum in non-ferrous metals. Zinc can be cast with a variety of metals into alloys with excellent physical and chemical properties due to its good ductility and wear resistance [1]. At present, zinc is widely used in the field of galvanizing because of its good corrosion resistance [2]. China is a major producer and consumer of zinc. According to statistics, China’s refined zinc output in 2021 reached 6.561 million tons [3]. However, the basic storage of zinc mines in China is limited, about 44 million tons [3]. Thus, it is very necessary to develop technologies of recovering zinc from secondary resources.

More than 50% of China’s zinc is used in the galvanizing industry [4]. After that, scrap galvanized steel sheets are mainly recycled through a converter steelmaking process and electric arc furnace steelmaking process. The zinc enters the dust removal system with the flue gas during the steelmaking processes due to the low boiling point of zinc, resulting in a higher zinc content in the dust.

The steel industry has provided the most basic raw materials for China’s infrastructure construction in the past several decades. The production of China’s raw steel has consistently ranked first in the world since 2000, which has made outstanding contributions to China’s economic development. However, it also produces a large amount of dust at the same time. Approximately 25–30 kg of steel mill dust (SMD) are produced during the production of one ton of steel [5]. In 2021, the production of China’s raw steel reached 1.035 billion tons, which means that the production of SMD reached about 25–30 Mt/year [3,5].

A large amount of steel mill dust (SMD) is produced in steel plants every year. The SMD with a zinc content of less than 1% can be returned to the ironmaking and steelmaking process. However, SMD with high zinc content, such as converter dust and electric arc furnace (EAF) dust, as shown in Table 1, cannot be returned to the ironmaking process due to the presence of K, Na and Zn [6]. The existence of these elements will seriously damage the normal operation of a blast furnace (BF). It has been previously determined that zinc will produce a “zinc cycle” in the BF, causing the gas permeability of the BF material column to drop sharply. Moreover, zinc is easily deposited in the gap of the coke column skeleton, destroying the coke strength [7,8]. Most of the SMD with high zinc content is disposed of by landfills, which not only takes up lots of land, but causes great damage to the local environment [9]. Harmful components in the dust will change the soil composition near the storage yard and cause soil pollution [10]. Moreover, the existence of heavy metals and trace radioactive elements in the dust will pose a great threat to the local water safety [11]. Additionally, its fine-grained particles are easily dispersed by wind, which will not only reduce visibility, but also pose a great threat to human health. Furthermore, the stacking of SMD with high zinc content causes a huge waste of resources. Thus, removing zinc in SMD is of great significance to the recovery and utilization of SMD.

The aim of this study is to review recovery processes of SMD, especially the SMD with high zinc content. The physical and chemical properties of SMD are introduced firstly. Then, the methods and current situation of recovering valuable metals from SMD are introduced. Lastly, technical challenges of extracting valuable metals from SMD are summarized, and corresponding solutions are described, aiming to provide useful guidance on the sustainable utilization of steel dust.

## 2. Sources and Characteristics of SMD

**Figure 1:**
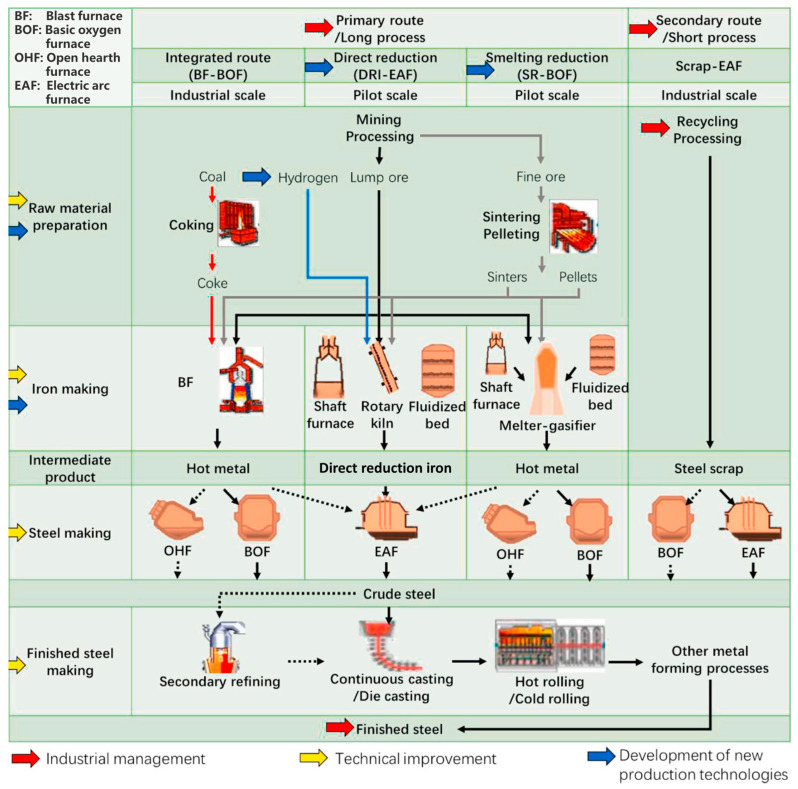
Typical steelmaking procedures [12].

There are many kinds of SMD generated in various equipment of the steel production process, including sintering machine, blast furnace, converter and electric arc furnace, as illustrated in Figure 1 [12]. It can be divided into three categories according to its composition. SMD with low zinc and low alkali metal content, such as BF gravity dust ash and iron oxide scale, can be directly used in the sintering process and returned to the ironmaking process. SMD with low zinc and high alkali metal content, such as electrostatic precipitator ash in the sintering process, can be used as raw materials for potassium salt production. In addition, returning SMD with medium or relatively high zinc content, such as BF bag ash, converter dust and EAF dust as shown in Table 1, to the sintering process will cause cyclic enrichment of zinc, which poses a threat to the smooth operation of the BF.

**Table 1:** Chemical composition of SMD [8,13].

Classification	Chemical Composition (wt.%)					Zinc Oxides
	TFe	C	Zn	Mg	Alkali Metal	
BF dust	23.4–35.6	18.5–33.0	1.2–3.4	0.5–1.1	0.4–1.9	ZnO, ZnFe_2_O_4_
Converter dust	50.0–80.0	-	1.7–9.4	0.4–2.2	-	ZnO, ZnFe_2_O_4_
EAF dust	29.0–48.6	0.4–3.0	3.8–27.0	0.4–2.2	1.2–2.7	ZnO, ZnFe_2_O_4_

**Figure 2:**
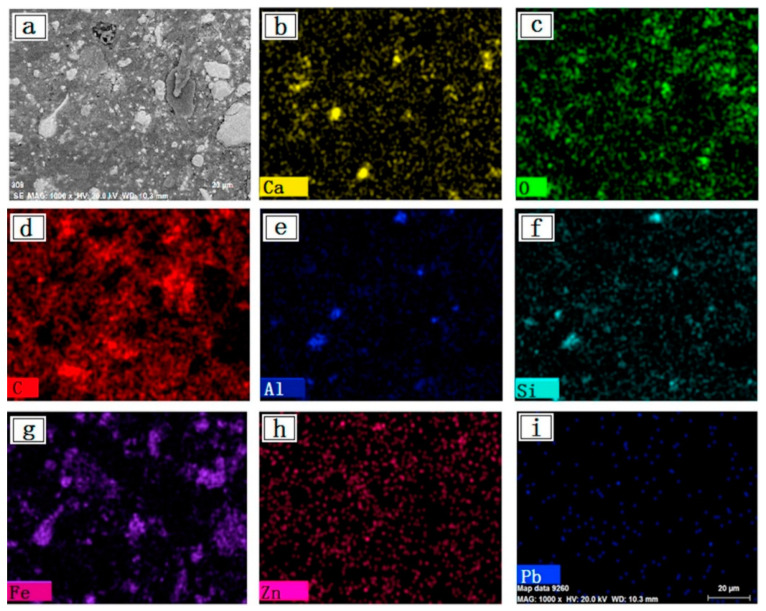
SEM image and element distribution of BF dust, (**a**) SEM; (**b**) Ca; (**c**) O; (**d**) C; (**e**) Al; (**f**) Si; (**g**) Fe; (**h**) Zn; (**i**) Pb [13].

In the ironmaking process by a BF, the zinc in BF dust mainly comes from sintered ore [5]. Zinc is a trace element in the raw materials of the BF, and it usually enters the BF in the form of oxides and sulfides. Zinc compounds are easily reduced under high temperature and reducing atmosphere, and then vaporize at high temperature due to their low boiling point, entering the flue gas and rising accordingly. A small amount of zinc will deposit after oxidation in the low temperature area of the upper part of the BF, then it will fall with the furnace charge. This part of zinc will cause the cyclic accumulation of zinc in the BF, which is why the zinc content in the BF raw materials should not exceed 1% [5]. Most of the zinc enters the gas dust removal system with the flue gas and eventually enters the dust. Bag dust removal is a kind of fine dust removal method and it has become a common method due to its advantages of high dust removal rate, low cost and low pollution. As shown in Table 1, the total iron content of this dust is 23.4–35.6%, and the iron mainly exists in the form of magnetite. Its carbon content is 18.5–33.0%, which can reduce the amount of carbon used for fire recovery. The zinc content is 1.2–3.4%, and Zn mainly exists in the form of ZnO and ZnFe_2_O_4_. Figure 2 is a SEM image and shows element distribution of BF dust. It can be found that the position of zinc element is not related to other elements, and the dispersed composition of certain minerals in zinc blast furnace dust [14]. BF dust has a small particle size and a light specific gravity. Generally, the cumulative pass rate of BF dust is 50–65% when using a 200-mesh sieve. Therefore, it is easily dispersed in the atmosphere and severely pollutes the surrounding environment.

**Figure 3:**
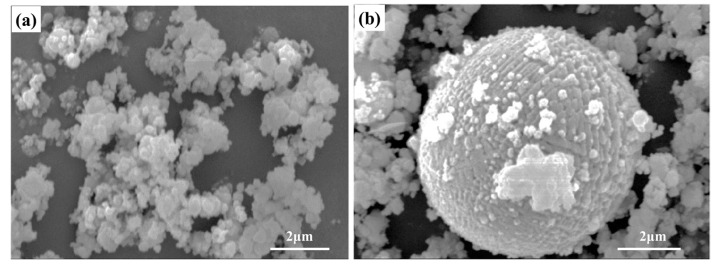
SEM of EAF dust particles: (**a**) irregular agglomerates; (**b**) larger molten spheres [16].

In the steelmaking process by an EAF, the zinc in EAF dust mainly comes from galvanized scrap. Figure 3 is a SEM image of EAF dust. It can be seen that it is composed of agglomerates of very fine irregular and slightly spherical particles (Figure 3a) and some obviously melted larger spherical particles (Figure 3b). The shape of these particles reflects the generation mechanism of EAF dust in the steelmaking process [15]. Studies have shown that the main reason for the formation of EAF dust is the rupture of the CO bubbles on the melt surface, which breaks the melt film that originally covered the bubbles in small droplets, and then these small droplets will also be discharged with the flue gas. In addition, due to the rapid heating and violent agitation in the EAF, the metal with low melting point will be evaporated or brought directly into the dust removal system by the hot air stream together with some oxides in the slag. The above furnace dust is finally deposited in the dust removal system to form EAF dust. According to literature reports, in China, the iron content in EAF dust is about 29.0–48.6%, and the zinc content is about 3.8–27.0%, as shown in Table 1. It is easy to cause soil alkalinity when EAF dust is piled up due to its alkaline composition. In addition, EAF dust contains heavy metal elements such as Zn, Pb, Ni and Cr. These elements will seep out after being washed by rain during stacking, causing heavy metal pollution of water and soil resources.

## 3. Processes for Recovering Metals from SMD

For about three decades, the resource utilization of SMD has become a research hotspot in the metallurgy field. Numerous processes have been developed to recover metals from SMD. These processes can be divided into the following three categories: physical process, hydrometallurgical process and pyrometallurgical process [17].

### 3.1. Physical Process

The physical processes can divide the steel dust into a high zinc part and low zinc part, which mainly depends on the particle size, density and magnetism of different minerals. The physical processes mainly include mechanical separation, hydro-cyclone dezincification and magnetic separation [16].

Mechanical separation can be divided into dry and wet processes. The mechanical separation methods mainly utilize the phenomenon that zinc is generally enriched in fine particles [18]. They can use centrifugal force or gravity to separate materials with different particle size and density. After mechanical separation, the coarse powder can be used in the ironmaking process, and the fine powder with high zinc can be further processed and utilized.

A hydro-cyclone is a type of wet particle size classification equipment, which is the main equipment for hydro-cyclone zinc removal [19]. This method uses the centrifugal force inside the hydro-cyclone to separate the mud with different particle size grades. The mud can be divided into a fine-grained high zinc part and coarse-grained low zinc part by the centrifugal force as the zinc in steel dust is generally found in fine particles [20]. The low zinc part can be reused in the sintering process after treatment, and the high zinc part can be used as raw material for zinc smelting.

Magnetic separation can separate different substances in SMD by different magnetic permeability of minerals. It is necessary to remove carbon from the SMD using flotation methods to improve the efficiency of magnetic separation [21]. This method mainly utilizes the characteristic that zinc is generally accumulated in dust with low magnetism [19]. After magnetic separation, the magnetic dust can be used as raw material for ironmaking.

The physical process is simple and easy to operate while, generally speaking, the efficiency of this method is low and the removal rate of zinc is low. Thus, it is only the pretreatment method of the hydrometallurgical process or pyrometallurgical process.

### 3.2. Hydrometallurgical Process

**Figure 4:**
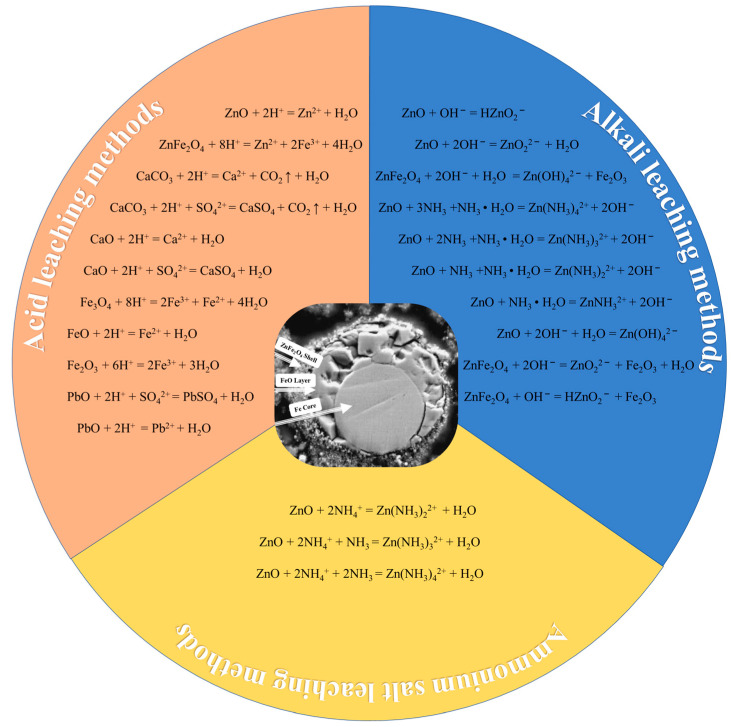
Main chemical reactions in hydrometallurgy process [22,23,24,25] (the internal picture is a SEM image of an SMD particle [26]).

The hydrometallurgical processes are mainly used to recover zinc from the SMD with medium or high zinc content. Generally, the hydrometallurgical processes can be divided into acid leaching methods, alkali leaching methods and ammonium salt leaching methods [22,23]. The main chemical reactions in the hydrometallurgy process are shown in Figure 4.

#### 3.2.1. Acid Leaching Methods

**Figure 5:**
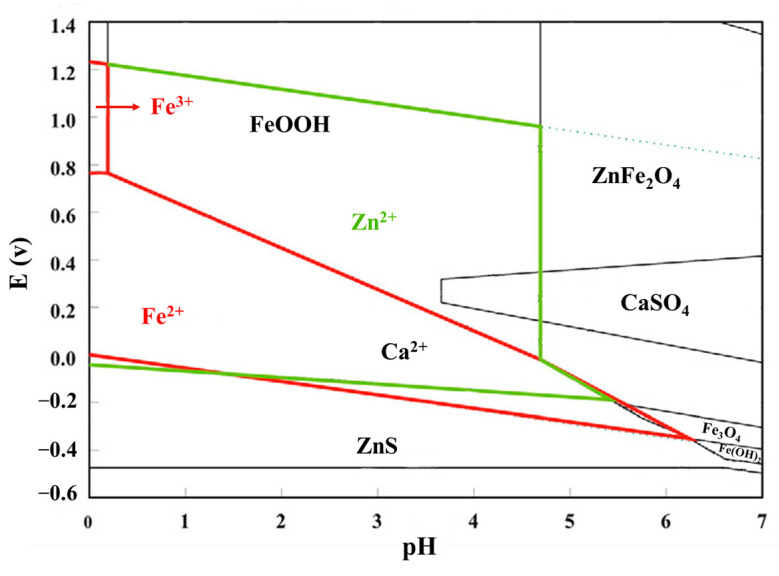
E–pH diagrams for the Zn–Ca–Fe–S–H_2_O system at 20 °C [27].

In recent years, many acid leaching methods have been investigated to recover valuable metal elements from SMD. In the acid leaching process, the leaching reagents mainly include hydrochloric acid and sulfuric acid. The key to metal recovery from SMD by these methods is the dissolution of elements in acidic solution, as shown in Figure 5.

Many studies have shown that concentrated acid used as a leaching agent can simultaneously leach zinc and iron from SMD with high zinc content. When dilute acid and weak acid are used as leaching agents, the leaching rate of zinc is very low. The analysis of the leaching residue shows that zinc in ZnO is easily leached by acid, but ZnFe_2_O_4_ is rarely dissolved. Therefore, the structural destruction of ZnFe_2_O_4_ is the key to whether zinc in dust can be efficiently extracted.

Zinc in SMD can be leached selectively only by controlling the concentration of leaching solution and pH value. Trung et al. used sulfuric acid to leach zinc from converter dust [23]. Seventy percent of zinc in the dust is leached into the solution under optimum reaction conditions. However, a small amount of iron enters the solution at the same time. Then, they found that the iron in the solution can be removed by adjusting the pH of the solution. Kukurugya et al. used sulfuric acid to leach zinc from EAF dust [27]. When the concentration of sulfuric acid was 1 mol/L, the ratio of liquid to solid was 50 and the reaction temperature was 80 °C, the leaching rate of zinc could reach 87%.

Studies have shown that hydrochloric acid used as a leaching agent can achieve high-efficiency leaching of zinc from dust, but iron and other metal elements were also leached at the same time [28,29]. NÚñez et al. used 0.5–1 mol/L HCl solution to extract zinc from ZnFe_2_O_4_ at 90–100 °C [30]. When the leaching time was 2 h, the leaching rate of zinc was higher than 90%, and the leaching rate of iron was 8%.

Zinc in dust can also be leached out by organic acids. Zhang et al. found that when the BF dust was leached by 0.2 mol/L iminodiacetic acid at 20 °C with a liquid–solid ratio of 10 for 2 h, the leaching rate of zinc in the dust could reach 62.78% [6]. Zinc in the leaching solution can be separated by adding a chemical precipitator.

Furthermore, some relatively mature zinc recovery processes by acid leaching have been developed, such as ZINCEX and Rezade. The ZINCEX treatment process mainly includes three steps: leaching, extraction and back extraction [26]. Firstly, zinc and cadmium oxides and halides are leached with sulfuric acid at 40 °C, and the leaching solution is neutralized by lime or limestone to remove iron and aluminum to obtain a qualified zinc-containing net solution. Secondly, the P204 extractant made by mixing a Di-(2-ethylhexyl) phosphoric acid (D2EHPA) and kerosene solution under the condition of pH = 2.5 is used to extract and enrich the zinc in the purified liquid. Finally, the organic phase containing zinc is washed with water to obtain a qualified electrolytic zinc solution, which is sent to the electrolysis workshop. The cadmium is recovered from the purification residue, the lead is recovered from the leaching residue, and the electrolytic waste liquid can be returned to the leaching process. This method has been put into operation in northern Spain and can process 80,000 tons of electric furnace dust annually.

The Rezade treatment process is an acid leaching method developed in France to treat zinc-containing SMD. It mainly includes three steps: leaching, purification and electrodeposition. First, strong acid solution is used to leach out the zinc, lead and cadmium elements in SMD. Then, the lead and cadmium in the leachate can be replaced by zinc through adding zinc powder to the filtered solution. After that, the solution is filtered to obtain an electrolytic liquor that can produce zinc. The zinc powder can be transferred from the electrolyte to the product by the electrodeposition method, and the purified slag and leaching slag can be returned to the steelmaking process to recover valuable metals such as iron.

#### 3.2.2. Alkali Leaching Methods

**Figure 6:**
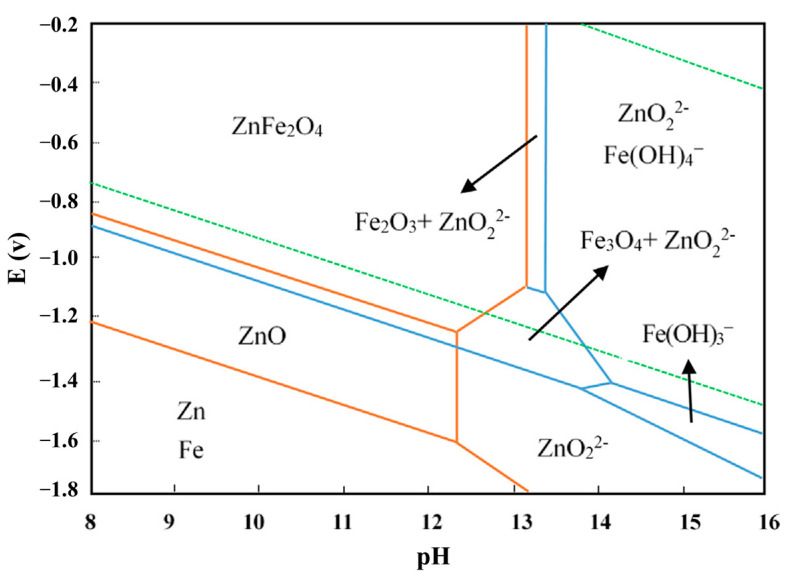
The E-pH diagrams of Fe-Zn-H_2_O system at 200 °C [31].

Zinc oxide is an amphoteric oxide, which can be dissolved not only in acidic solution, but also in alkaline solution, as shown in Figure 6 [31]. Dutra et al. found that when NaOH solution was used to treat EAF dust, ZnO in the dust can be easily dissolved, while zinc ferrite remained in the solid residue [5]. Xia et al. [24] used a microwave power of 2.45 GHz as an aid in the process of leaching zinc from EAF dust by 8 mol/L NaOH solution. Although the leaching rate of zinc was greatly increased after microwave heating, the zinc in ZnFe_2_O_4_ still could not be leached, resulting in a low leaching rate of zinc. Many researchers have added an alkali fusion step before the alkali leaching to destroy the structure of ZnFe_2_O_4_, in order to achieve the selective separation of zinc and iron in steel dust. Zhang et al. [31] found that zinc in zinc ferrite can be selectively leached by a hydrothermal reduction method in a NaOH system, and 70% zinc in zinc ferrite can be extracted by this method.

Ural Institute of Technology alkaline leaching method is a process developed by the Ural Institute of Technology to extract zinc from BF dust. Firstly, concentrated NaOH solution is used to leach zinc from zinc-containing dust, and the silicon element in the dust also enters the leachate in the form of ions at the same time. Secondly, liquid–solid separation is carried out after dilution with water. Then, CaO is added to the filtrate to remove silicon from the filtrate to obtain a solution containing a high concentration of Zn(OH)_2_. Finally, Zn(OH)_2_ is obtained by evaporation and crystallization and filtration of the solution, and ZnO product is obtained after Zn(OH)_2_ is dehydrated.

#### 3.2.3. Ammonium Salt Leaching Method

Zinc oxide can be dissolved in ammonium salt solution, as shown in Figure 4. Thus, ammonium salt solution can also be used to treat zinc-containing SMD.

Miki et al. [25] used calcium oxide to pretreat electric furnace dust. It can convert zinc ferrite in electric furnace dust into ZnO and Ca_2_Fe_2_O_5_. The halogen elements and heavy metals in the dust can also be removed by this method. Then, NH_4_Cl solution was used as leaching solution for selective leaching of zinc. The zinc in the dust treated with CaO was almost completely leached out after leaching by 2 mol/L NH_4_Cl solution at 70 °C for 2 h, and the leaching rate of calcium was only about 20%.

The EZINEX process invented by Italy mainly includes process steps such as salt solution leaching, filtration, purification and electrodeposition. The process uses a mixture of NH_4_Cl and NaCl as the leaching agent, and the leaching temperature is usually in the range of 70–80 °C [27]. The main reaction equation is as follows:

ZnO + 2NH_4_Cl = Zn(NH_3_)_2_Cl_2_ + H_2_O. (1)

At the same time, Pb, Cu, Cd and other elements in the dust will also be leached. After that, the filtered leaching residue mixed with carbon as a reducing agent can be returned to the steelmaking process. Pb, Cu and Cd can be replaced by adding zinc powder to the leaching solution. The zinc can be recovered from the purified solution by electrolysis with a titanium plate as the cathode and graphite as the anode. The waste electrolyte can be used as secondary leaching solution.

Table 2 is a summary of the advantages and disadvantages of various hydrometallurgical methods. When H_2_SO_4_ or HCl solution was used as a leaching agent, it could achieve efficient leaching of zinc from SMD. However, iron in SMD would be leached at the same time, which required an additional complex iron removal process. The leaching process using concentrated acid as a leaching agent required the equipment to have good acid corrosion resistance. Dilute strong acid and organic weak acid used as the leaching agent had a very low extraction rate of zinc from SMD due to their inability to extract Zn in ZnFe_2_O_4_. The result of zinc extraction by the alkaline method is similar to that of dilute strong acid and weak acid, and the Zn in ZnFe_2_O_4_ could not be leached either. The leaching slag cannot be reused in a steel mill due to its high residual content of zinc. The alkali fusion–alkali leaching method can convert ZnFe_2_O_4_ into ZnO, Na_2_ZnO_2_ and Fe_2_O_3_ through the alkali fusion process, and then zinc in the ZnO and Na_2_ZnO_2_ can be leached out by the alkali leaching method, so as to achieve efficient and selective leaching of zinc. However, this method requires a large number of alkaline substances, and the high temperature required during the calcination process results in high energy consumption. In the subsequent treatment of the leachate, a large amount of acid is required for neutralization.

### 3.3. Pyrometallurgical Process

Pyrometallurgical processes are an effective way to recycle elements from SMD. At present, the recovery process of steel dust is mainly divided into two categories: direct reduction method and smelting reduction method. The temperature of the reaction zone of these two methods is different, which makes the reaction of the raw materials in the furnace different, resulting in different products.

The zinc, lead and other low-boiling metal oxides in the dust are reduced to metal vapor and separated from the residue by these two methods. The difference is that the temperature in the reaction zone of the direct reduction method is lower, the residue is not melted and the iron oxide in it is reduced to direct reduced iron. The temperature in the reaction zone of the smelting reduction method is very high, and almost all the metal oxides in the dust are reduced and smelted. Commonly used direct reduction methods include the Waelz process and rotary hearth furnace process. The smelting reduction methods include OxyCup, DK process and coke-packed bed process. The characteristics of these processes are shown in Table 3. These processes are described in detail below.

#### 3.3.1. Waelz Process

**Figure 7:**
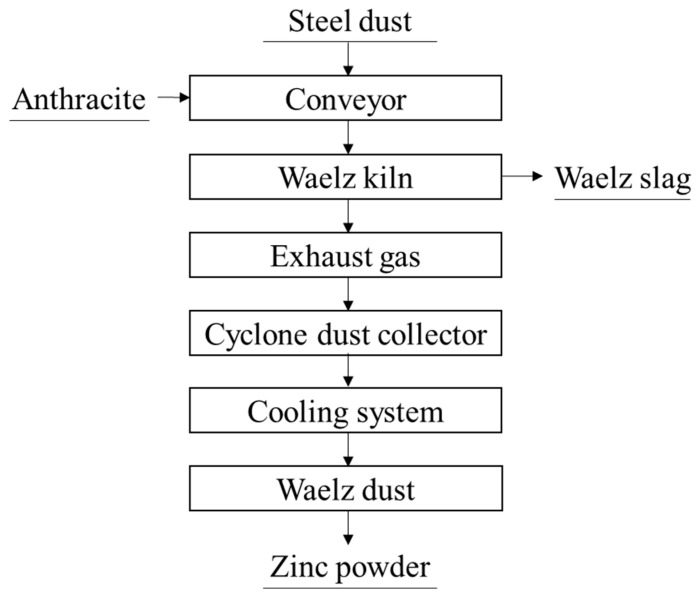
The Waelz process flow [8,13,36].

The Waelz process is currently the most widely used method for recovering dust containing medium levels of zinc [8]. Figure 7 is a simplified flow chart of the Waelz process. Initially, the steel dust, reductant and flux are mixed evenly to make balls. The granulated material is sent to the Waelz kiln for drying and preheating. At the reaction temperature of 1000–1200 °C, the iron oxide in the dust is reduced to metallic iron, and the zinc volatilized in the form of metal vapor. The zinc in the gas can be oxidized into a solid, and then it will be recovered by controlling the air from the outlet of the Waelz kiln. The metallic iron in the charge is re-oxidized to form a by-product called Waelz slag [36].

The Waelz process has the advantages of large treatment capacity and relatively good economic benefits. However, there are some disadvantages of this method that cannot be ignored. The Waelz process requires that the content of zinc in the dust should be higher than 16% to ensure economics of the process [13]. To prevent ring formation in the furnace, it is necessary to add a flux to the raw materials, which leads to an increase in the gangue content in the slag and increases the output of secondary solid waste. Moreover, the iron in the residue is not sufficiently enriched due to the presence of residual zinc and lead [37]. In addition, it needs a lot of energy consumption to keep the high reaction temperature in Waelz kiln, which increases the cost.

#### 3.3.2. Rotary Hearth Furnace (RHF) Process

**Figure 8:**
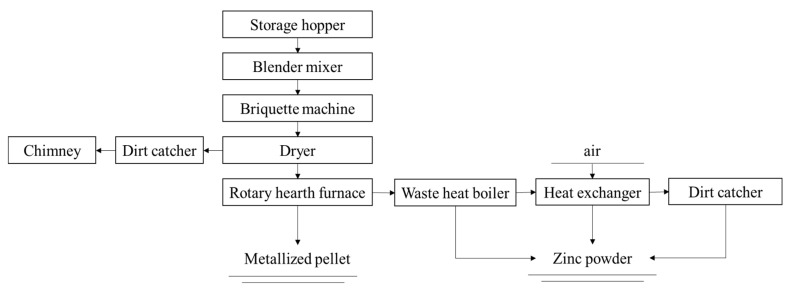
Flow chart of RHF process [32,38,39].

The RHF process is widely used to recover SMD containing zinc and lead. This process is mainly composed of five parts: proportioning, ball making, direct reduction, flue gas treatment and dust recovery [32]. Figure 8 is the flow chart of the RHF process. Steel dust, reductant (carbon powder) and binder are mixed together to form pellets. They are evenly arranged in the RHF after drying with a material layer of 1~3 pellets in height. Then, the temperature in the furnace is raised to reduce the metal oxides in the dust. The pellets are directly reduced at 1250~1350 °C for 10~20 min to obtain the direct reducing iron, which can be returned to the steel works for smelting after treatment [38]. The zinc and lead oxide in the steel dust are reduced to metal vapor and discharged from the RHF together with the flue gas. When the flue gas passes through the cooling system, zinc vapor meets air and then oxidizes into fine zinc oxide particles. Finally, 40~70% of coarse zinc oxide dust can be obtained from the dust collector.

The RHF direct reduction method is considered to be an effective method to treat EAF dust containing a large amount of iron, carbon, zinc and alkali. The products of this process are high-grade zinc and direct reduced iron. This process may have more commercial and environmental advantages than the Waelz process. The RHF device has the advantages of simple operation and maintenance, high working efficiency and strong processing ability. However, there are still some technical problems and shortcomings that have not been overcome [39], such as low energy efficiency, large equipment area and high investment. Therefore, this process needs to be optimized to improve its economic benefits.

#### 3.3.3. OxyCup Process

**Figure 9:**
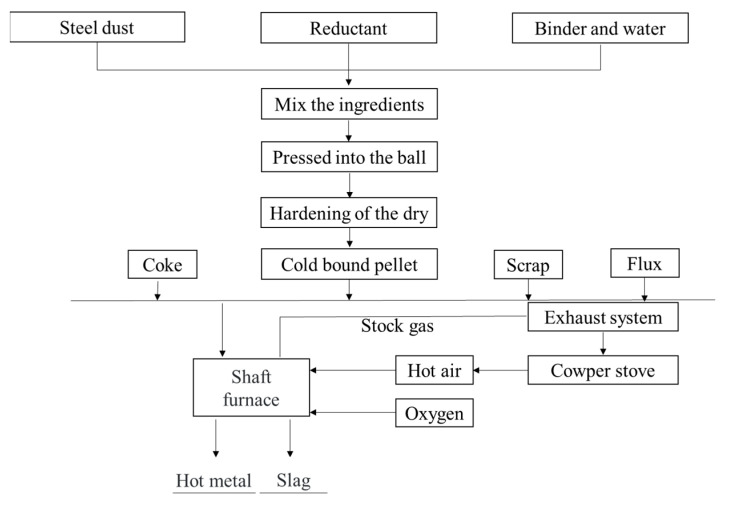
Flow chart of OxyCup process [33,40,41,42].

The OxyCup process is now a representative smelting reduction method of the pyrometallurgical process, which can be used to realize the recovery and utilization of zinc-bearing SMD. Figure 9 is the flow chart of the OxyCup process. The main device of this process is a large oxygen-enriched cupola, which can be used to recover the fine particle dust, scrap steel and scrap iron generated in the traditional iron- and steelmaking process [33]. The raw materials used in this process must be briquetted to meet the strength requirement of self-reducing block [40]. All kinds of waste materials from iron and steel plants are mixed, pressed into blocks and added into a shaft furnace after pretreatment [41]. The reduction of iron oxide in the shaft furnace starts when the temperature of the reaction zone rises to 900 °C. Then, the sponge iron is generated with the temperature rising to 1400 °C [42]. Finally, the reduced iron and slag are melted into a liquid state at high temperature and then flow out from the shaft furnace through a siphon system. Flue dust generated in this process will be gathered into the filter. The product of this method is hot metal, which can be used in the steelmaking process. Moreover, the high calorific value of gas, dust with high zinc content and other by-products are obtained at the same time.

The OxyCup process has the function of high temperature smelting and reduction, which makes it have no special requirements for zinc content in raw materials. It can make up for the defects of the Waelz process [33]. Moreover, this method is an environmental protection method to recover the steel dust due to its low pollution emission. However, the reduction mechanism of the steel dust in the furnace is still unclear. Thus, the reduction process in the furnace should be further studied to achieve better furnace operation.

#### 3.3.4. DK Process

**Figure 10:**
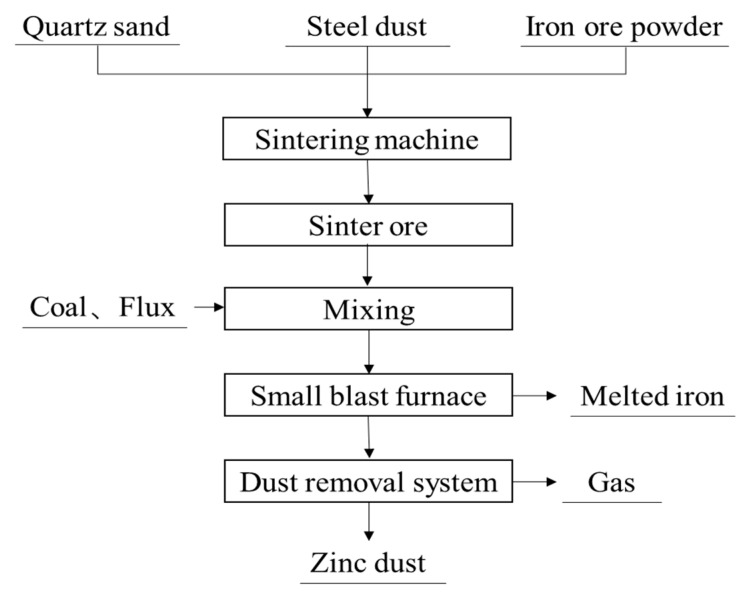
Flow chart of DK process [34].

The DK process has been applied by DK Company in Germany. Its smelting principle is the same as that of a traditional BF [34]. The DK process flow chart is shown in Figure 10. The raw materials of this process are converter dust, quartz sand and coarse iron ore powder. These raw materials are first mixed evenly, then agglomerated in the sintering process and finally smelted in the furnace. Quartz sand is used to adjust the basicity of slag, and a small amount of coarse iron ore powder is added to improve the permeability of the sintering material layer.

This method is mature in technology and easy to operate. Moreover, the idle small BF and sintering machine in the steel plant can be used as the main equipment of the DK process, thus saving the equipment investment. However, this method needs to consume a lot of fuel to ensure high temperature in the furnace. In addition, the high zinc and alkali load in the furnace will inevitably impede the operation of the furnace [34].

#### 3.3.5. Coke-Packed Bed Process

**Figure 11:**
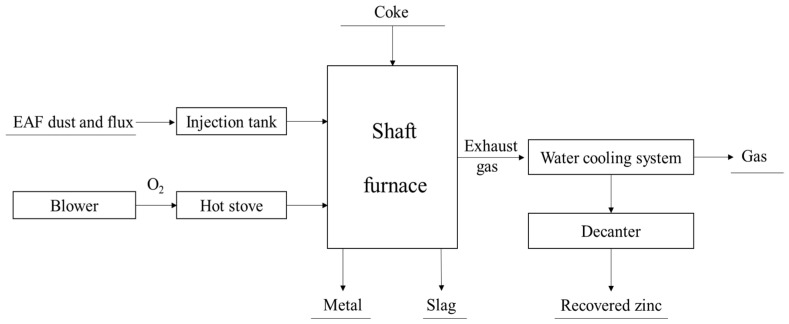
Flow chart of coke-packed bed process [35,43,44,45].

The coke-packed bed process is a melting–reduction process developed by Kawasaki Steel Corp, which is used to recover zinc and iron from EAF dust [35,43,44,45]. Figure 11 is the flow chart of the coke-packed bed process. The main equipment of this process is a shaft furnace with coke-packed bed, which has two-stage tuyeres. The powdery raw material is injected through the upper tuyere, and then melted immediately in the raceway. There is a high temperature and intensively reductive region between the upper and lower tuyeres to ensure good heat exchange between the falling coke and the rising gas. The metal oxides in the raw materials can be easily reduced to metals when they pass through the coke-packed bed at high temperature. The metals with high boiling point will drop into the hearth together with the slag, and the metals with low boiling point, including zinc and lead, will evaporate from the top of the furnace. The main products of this method are molten metal, crude zinc oxide and slag.

The coke filling method can effectively separate the metals in the dust, and these products can be used as raw materials for the production of iron and zinc. However, this method needs a lot of fuel to make the temperature of the lower tuyere higher than 1550 °C to ensure the reduction of metal oxides. In addition, the furnace gas pressure and top gas temperature should be ensured to prevent zinc and lead from adhering to the furnace wall.

Pyrometallurgical processes can recover iron and zinc from dust to some extent, but there are some problems in these processes that need to be solved urgently [46,47,48]. Specifically, the Waelz process has high requirements for raw materials, an unstable production process and high energy consumption. The RHF process has low production efficiency and high investment cost. The OxyCup process has a short equipment operating cycle and high cost due to the use of coke. The DK process has high energy consumption, and the smoke from the sintering process will pollute the environment. The coke-packed bed process has high energy consumption and short service life of equipment.

### 3.4. Pyrometallurgy–Hydrometallurgy Combined Treatment (PHCT) Processes

Combining pyrometallurgical and hydrometallurgical processes to treat SMD can realize the comprehensive recovery of various valuable metal elements in SMD. The pyrometallurgical part of the PHCT process can use an RHF to directly reduce and roast steel dust, in which metals with lower boiling points such as zinc, Pb and Cd will be reduced into the flue gas. After cooling, the flue gas is collected by the bag collector, and then the alkali metal in the bag dust is recovered by the water immersion method. Afterwards, the Zn, Pb and Cd in the leaching residue can be extracted by ammonia leaching.

This method can destroy ZnFe_2_O_4_ at high temperature and achieve high-efficiency separation of zinc and iron in SMD. Zinc mainly exists in the form of ZnO that is easily leached after the SMD is treated, so the recovery rate of Zn in the dust can be improved. The PHCT process can combine the advantages of pyrometallurgy and hydrometallurgy methods. It overcomes the low extraction rate of zinc by hydrometallurgy methods and the low purity of zinc by pyrometallurgy methods. However, this process involves more operating steps and higher investment costs.

The resource utilization of SMD may be realized through physical, hydrometallurgical and pyrometallurgical processes, and the environmental problems caused by dust storage can be also solved at the same time. The advantages and disadvantages of these treatment processes are compared in Table 4.

## 4. Discussion

### 4.1. Conclusions

As the primary mineral resources of zinc continue to decrease and the demand for galvanized steel continues to increase worldwide, the recovery of secondary zinc resources is becoming increasingly important. The recovery of valuable metals from SMD can not only reduce the environmental protection pressure of steel plants, but ease the pressure on mineral resources and bring economic benefits.

The physical methods divide the dust into zinc-rich dust and low-zinc dust according to the difference in particle size and density. Among them, the low-zinc dust can be used in the sintering process and returned to the ironmaking and steelmaking process. However, the zinc content in the zinc-rich dust is not enough to be directly used in the production of zinc metal, and further enrichment of zinc is required. Therefore, the physical method can only be used as a pretreatment process for the hydrometallurgical process and the pyrometallurgical process.

The hydrometallurgical processes can obtain valuable metals with high mass fraction. Energy consumption of the treatment processes is low due to them being carried out at a low temperature. However, these methods require high corrosion resistance of the equipment. Leaching slag cannot be used as ironmaking raw material of BF smelting due to its complex composition, and it cannot meet the storage requirements of environmental protection law.

Pyrometallurgical processes are nowadays the main methods to recover the SMD. The efficiency of these methods is relatively high, and the valuable elements in the dust can be cyclically enriched. Moreover, the products of these methods are hot metal or metalized pellets, which can be directly returned to the steelmaking process. However, these pyrometallurgical processes need further improvement due to high energy consumption, low purity of products and large equipment.

The residue after the pyrometallurgical treatment in the combined process of PHCT can be returned to the ironmaking and steelmaking process due to its high degree of metallization and low zinc content. Afterwards, other valuable metals enriched in the dust can be gradually extracted through hydrometallurgical processes to achieve full composition recovery of metal elements in SMD. This process is suitable for the recycling of a large variety of zinc-containing dust since it is suitable for most zinc-containing dusts. However, the technological process also has disadvantages that cannot be ignored, such as being more complicated, more operating steps and higher investment costs.

### 4.2. Prospects

The compositional characteristics of SMD in China are mainly high iron and low zinc. Thus, only recovering zinc from SMD is not only uneconomical, but also a huge waste of iron resources. Recovering zinc in steel dust by the hydrometallurgy process has low economic benefits, and the residue after leaching is difficult to reuse in steel plants, resulting in a waste of iron resources. It is also difficult to economically recover the zinc in SMD using the Waelz process due to the low zinc content in SMD. Therefore, it is very necessary to realize the synchronous recovery of zinc and iron from SMD. The pyrometallurgical process and the pyrometallurgy–hydrometallurgy combined treatment process are the main trends in the recycling of SMD.

In order to realize the economic recovery of valuable metals in SMD, it is necessary to solve the problems of complex structure and correlation of metal-containing phases, re-oxidation of non-ferrous metals after reduction and enrichment of harmful impurities in the generated residues. These problems require further investigation of reactions of the phases in SMD on the basis of thermodynamics and kinetics, and additional processing of the secondary solid waste.

## Figures and Tables

**Table 2:** Comparison of different hydrometallurgical leaching processes [22,24,25,28].

Classification	Leaching Agent	Advantages	Disadvantages
Acid method	H_2_SO_4_	(1) Concentrated sulfuric acid can achieve high-efficiency extraction of zinc (2) Dilute sulfuric acid can achieve selective extraction of zinc	(1) Concentrated sulfuric acid has poor selective leaching effect on zinc in dust, and iron in dust will be leached at the same time (2) Dilute sulfuric acid used as a leaching agent has low leaching rate of zinc in steel dust
	HCl	High-efficiency extraction of zinc can be achieved under certain conditions	The selective leaching effect of zinc in dust is poor, and Fe, Cu and Cd in steel dust will also enter the solution
	Acetic acid	Zn and Pb in dust can be selectively leached out	The leaching rate of Zn is low since zinc in ZnFe_2_O_4_ cannot be leached
Alkaline method	NaOH	Zn and Pb in steel dust can be selectively leached out	(1) Zinc in ZnFe_2_O_4_ cannot be leached out (2) The PbO in steel dust will also be leached
	Ammonia	Zn in steel dust can be selectively leached out	The leaching rate of Zn is low since zinc in ZnFe_2_O_4_ cannot be leached
Alkali fusion–alkali leaching method	NaOH-NaOH	(1) High leaching rate of zinc in steel dust (2) Zn in steel dust can be selectively leached out	(1) The process is complicated and there are many operation units (2) High consumption of NaOH (3) Large amount of waste liquid
	CaO-NH_4_Cl	(1) Can realize the effective separation of Zn and Fe (2) High leaching rate of zinc in steel dust	(1) The process is complicated and there are many operation units (2) High consumption of CaO (3) The high temperature of roasting pretreatment requires high temperature resistance of the equipment, and the investment cost is large

**Table 3:** Comparison of different pyrometallurgical processes [8,13,32,33,34,35].

Process	Products	Advantages	Disadvantages
Waelz	Zinc-rich dust; Waelz oxide	(1) Fewer operation steps and simple process (2) Well-established technology and reliable process	(1) High maintenance costs (2) Low productivity (3) High requirements for zinc content of raw materials (zinc content > 16 wt.%)
RHF	Zinc-rich dust; Direct reduction iron	(1) Well-established technology and reliable process (2) Fewer operation steps and simple process (3) High metallization rate of residue	(1) High initial investment and operating costs (2) High operating temperature requirements
OxyCup	Zinc-rich dust; Molten iron; Slag	(1) Many types of dust that can be recycled (2) High metal recovery rate (3) High added value of the product	(1) The operation cycle of the equipment is short (2) The smelting cost is high
DK	Pig iron; Zinc-rich dust	(1) Well-established technology and reliable process (2) Low equipment investment (3) The produced pig iron can be used in the casting process	(1) High energy consumption (2) Serious environmental pollution (3) Poor long-term operation safety
Coke-packed bed	Zinc-rich dust; Molten iron; Slag	(1) Separation of metallic iron and slag can be realized (2) The raw material does not need to be processed by agglomeration	(1) The heat consumption is large and the waste heat is difficult to recover (2) The material requirements of the equipment are high (3) The investment cost is high

**Table 4:** Comparison of several treatment processes of extracting metals from steel dust [16,22,46].

Treatment Process	Advantages	Disadvantages
Physical process	(1) Operation process is simple (2) Low investment	(1) Products cannot be recycled directly (2) Low efficiency
Hydrometallurgical process	(1) The leaching rate of valuable metals is high (2) High leaching efficiency	(1) The slag produced by leaching cannot be recycled (2) The amount of leaching agent is large (3) The treatment process is liable to cause pollution
Pyrometallurgical process	(1) Iron, carbon and other valuable elements in dust can be fully utilized (2) Products containing iron can be directly used as raw materials for ironmaking	(1) The metallization rate of the products varies greatly (2) The reaction process consumes a lot of energy
Combination of pyrometallurgical and hydrometallurgical process	(1) Combines the advantages pyrometallurgical and hydrometallurgical processes (2) High comprehensive metal recovery rate	(1) Many operation steps and complex process (2) High investment cost

## Data Availability

Data sharing is not applicable to this paper.
